# Decision aids linked to the recommendations in clinical practice guidelines: results of the acceptability of a decision aid for patients with generalized anxiety disorder

**DOI:** 10.1186/s12911-022-01899-2

**Published:** 2022-06-30

**Authors:** Vanesa Ramos-García, Lilisbeth Perestelo-Pérez, Amado Rivero-Santana, Wenceslao Peñate-Castro, Andrea Duarte-Díaz, Yolanda Álvarez-Pérez, María del Mar Trujillo-Martín, María Isabel del Cura-González, Pedro Serrano-Aguilar

**Affiliations:** 1 Canary Islands Health Research Institute Foundation, Tenerife, Spain; 2 Facultad de Ciencias de La Salud - Sección de Psicología, University of La Laguna (ULL), Tenerife, Spain; 3 Evaluation Unit (SESCS), Canary Islands Health Service (SCS), Tenerife, Spain; 4 Research Network On Health Services in Chronic Diseases (REDISSEC), Tenerife, Spain; 5 Network for Research On Chronicity, Primary Care, and Health Promotion (RICAPPS), Tenerife, Spain; 6 The Spanish Network of Agencies for Health Technology Assessment and Services of the National Health System (RedETS), Tenerife, Spain; 7 Research Network On Health Services in Chronic Diseases (REDISSEC), Madrid, Spain; 8 Network for Research On Chronicity, Primary Care, and Health Promotion (RICAPPS), Madrid, Spain; 9 Unidad de Apoyo a La Investigación, Gerencia Asistencial de Atención Primaria, Madrid, Spain; 10 Department Preventive Medicine and Public Health, University Rey Juan Carlos, Madrid, Spain; 11 Servicio de Evaluación del Servicio Canario de la Salud, Camino de Candelaria nº 44, Centro de Salud San Isidro-El Chorrillo 1ª planta, 38109 Tenerife, Spain

**Keywords:** Generalized anxiety disorder, Practice guideline, Patient decision aid, Acceptability, Shared decision-making

## Abstract

**Background:**

Generalized anxiety disorder (GAD) is one of the most prevalent mental health problems. Patients with GAD have unmet needs related to the information received about their disorder, its treatments and their participation in the decision-making process. The aim of this study is to develop and assess the acceptability of a patient decision aid (PtDA) for patients with GAD.

**Method:**

The PtDA was developed following the International Patient Decision Aid Standards. The recommendations of the Spanish clinical practice guideline (CPG) for patients with GAD were used as the basis. The first prototype was developed by an expert committee, further improvements were made with patients (n = 2), clinical experts (n = 13) and the project management group (n = 7). The acceptability of this second draft was assessed by patients non-involved in the previous phases (n = 11).

**Results:**

The final PtDA version included a brief description of GAD and its treatments. Most participants agreed that the PtDA was easy to use, visually appealing and useful. At least half of the participants learned new things about treatments and adverse effects.

**Conclusions:**

A PtDA was developed for patients with GAD based on recommendations from the Spanish CPG. It was improved and accepted by patients and clinical experts involved. An evaluation of its effectiveness on the shared decision-making process during the clinical encounter is planned.

**Supplementary Information:**

The online version contains supplementary material available at 10.1186/s12911-022-01899-2.

## Background

Anxiety disorders are among the most prevalent mental diseases. The World Health Organization (WHO) estimated that more than 260 million people in the world are affected by anxiety [1, 2]. Generalized Anxiety Disorder (GAD) is the most common anxiety disorder, with a lifetime prevalence of almost 4% [3]. GAD tends to be a chronic disorder, characterized by excessive and persistent worries about life issues, which may be accompanied by other mental and somatic symptoms [4]. GAD has a major impact on patients’ quality of life and implies direct costs for the health system and indirect costs for society (e.g., loss of productivity, anticipated mortality) [5, 6].

Treatment options for GAD include the use of drugs such as selective serotonin reuptake inhibitors (SSRIs), and serotonin and noradrenaline reuptake inhibitors (SNRIs), among others; and psychological interventions such as cognitive behavioral therapy or mindfulness [7, 8]. However, since there is no single best treatment appropriate for all patients with GAD, their preferences should be considered, along with treatment efficacy considerations, in the decision process. This preference-sensitive nature of GAD treatment indicates an ideal clinical scenario to apply a shared decision-making (SDM) approach, through which healthcare professionals and patients share healthcare information and make choices together [9]. The aim of SDM is to create a collaborative dialogue between these two agents, where patients’ values, preferences and concerns about the different available treatment options are discussed with the healthcare professional and incorporated into the decision-making process [10–12]. Interventions designed to promote SDM aim to improve decision-related variables such as patients’ objective knowledge about the disease and its available treatments, decisional conflict (i.e., uncertainty when choosing treatment), participation in decision-making, concordance between patients’ preferences and their final choice, or satisfaction with the decisional process [13, 14].

Research on SDM interventions in different health conditions has not shown unintended consequences of a more active participation by patients (e.g., increase in anxiety) [15, 16]. From a theoretical perspective, GAD specific characteristics such as persistent worry or intolerance to uncertainty could act like barriers to assume the responsibility of a greater involvement in the decision process [17, 18]. However, a recent study carried out by our team showed that 84.3% of GAD patients preferred an active or collaborative role in decision-making, although one third of the sample perceived more involvement than they desired [19]. These results reinforces the need to adequately assess patients’ preferences and perceptions of involvement, and to assure that decision-making is really collaborative for those who prefer it and does not impose them excessive responsibility.

For all of the above, it is necessary to develop resources to help GAD patients to make informed decisions that match their values and preferences about treatment. Among the resources proposed to facilitate SDM, Patient Decision Aids (PtDAs) stand out. PtDAs are developed in different formats (e.g., video, web, software, booklets) and include evidence-based information about a particular disease or health condition, about preventive measures or diagnostic tests, its available treatments, and the likelihood of their benefits and risks (preferably in a quantitative format). They also promote explicitly or implicitly the clarification of the patients’ own values, preferences and concerns about the potential consequences of the different treatments. Empirical findings have shown that PtDAs improve patients’ objective knowledge of treatment options, the accuracy of risk perceptions, decisional conflict, congruence between patients’ values and choices, participation in decision-making, satisfaction with the decision, and other variables related to the decisional process [16, 20].

In primary care settings, the availability of decision support tools based on the patients´ values and preferences are increasingly common [21]. Different studies have shown how the early involvement of patients and health professionals in the creation of support tools is useful for the development of PtDAs [21, 22]. However, despite the rapid growth in PtDA research, development and implementation observed over the last ten years; few studies have focused on anxiety disorders [23]. Furthermore, as happens with clinical practice guidelines (CPGs), one of the challenges of the PtDAs is that their development and updating require a rigorous systematic process, which can be time-consuming and costly [24].

Institutions such as the Cochrane Collaboration, the National Institute for Health and Care Excellence (NICE), the Spanish Network of Health Technologies Assessment Agencies (RedETS) and the Clinical Practice Guideline Library of the Spanish National Health System (Guisalud.es) have developed strategies that link the development of Clinical Practice Guidelines (CPG) to the development of PtDAs, in order to gain in developmental efficiency and improve decision making [25–27]. This strategy allows the early identification of the recommendations that are most sensitive to a SDM along CPG development [27, 28].

The present article describes the development process and acceptability testing of a web-based PtDA for patients with GAD. It was carried out in the context of the update of a CPG for the management of GAD in primary care [29] financed by the Spanish Ministry of Health within the annual work plan (2018) of the RedETS.

## Methods

### Setting for the development of a PtDA for patients with GAD in primary care

Generally, patients with GAD consult in the primary care setting and it is acceptable that they can be treated there. This context is optimal for SDM given the variety of treatment choices and resources available in the community [30]. In recent years, international and regional working groups have developed strategies linking the scientific evidence of CPGs and PtDAs in order to improve decision making and health outcomes [25–27, 31].

### Scope and purpose

The purpose of this PtDA is to promote SDM between patients and their healthcare providers, as well as patient involvement in their own care. The target population is any patient with GAD, independently of the time since diagnosis and treatment status. The content of this PtDA includes the description of GAD and the risks and benefits of its available pharmacological and psychological treatments, based on the scientific evidence identified in a CPG under concomitant development [29].

### Development of the digital PtDA

**Fig. 1:**
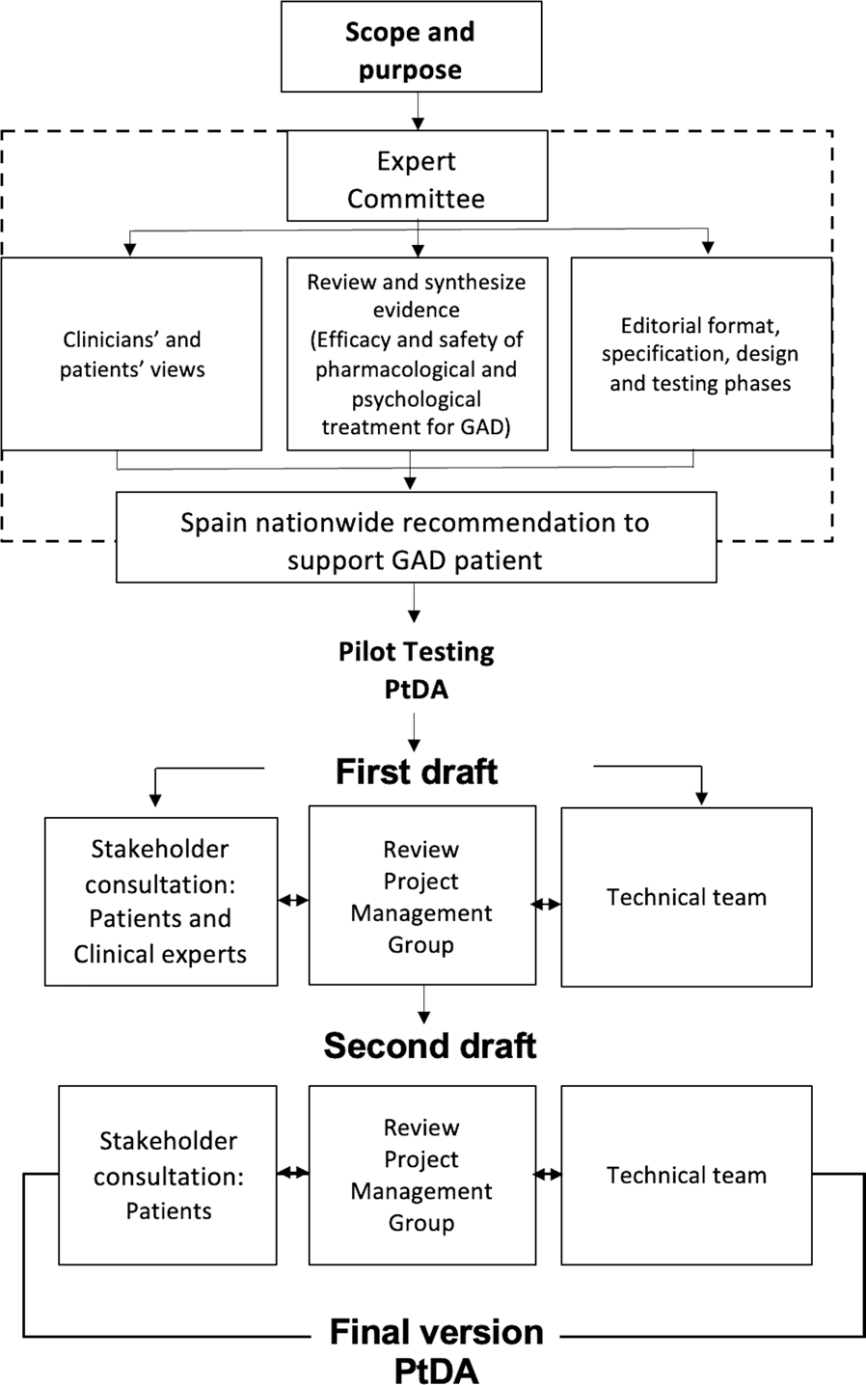
Development process of the decision aid. Adapted from Elwyn et al. [31]. The dotted box represents the earlier work for the development of the Spanish CPG recommendations for patient with GAD [64]

The development of the PtDA followed the methodological process proposed by Elwyn et al. [31] and the International Patients Decision Aid Standards (IPDAS) [20]. The process is summarized in Fig. 1. The empirical evidence on the efficacy and safety of pharmacological and psychological treatment for GAD was previously identified and synthetized as part of the update process of a CPG financed by the Spanish Ministry of Health [29], following the *Grading of Recommendations, Assessment, Development and Evaluation* (GRADE) methodology [32]. In addition, we carried out three different studies to explore the experiences, perceptions, preferences and information needs of GAD patients regarding the disease, their health care and available treatments: (1) a scoping review of qualitative studies [33]; (2) individual interviews with 8 GAD patients [34]; (3) a cross-sectional survey study with 70 GAD patients[19]. The results of this research were used to inform the PtDA content. The following working groups participated in the development of the PtDA.

#### Expert committee

An Expert Committee consisting of a multidisciplinary team composed by professionals specialized in primary care (medicine and nursing), psychiatry, psychology, mental health nursing, clinical pharmacology and social work, patients and methodologists (n = 13) formulated and graded the recommendations of the CPG. It used the EtD frameworks reported with the effectiveness and safety reviews, the primary study and consideration of resource use and costs.

#### Project management group

This group, integrated by methodologists, physicians and psychologists (n = 7), provided expert advice about the PtDA content, proposed implementation and dissemination strategy. Their opinion was considered throughout the PtDA development process and during the iterative review process.

#### Stakeholder consultations group

Thirteen clinical experts (2 primary care physicians, 3 psychiatrists, 5 psychologists and 3 nurses) and two patients with a current diagnosis of GAD reviewed the contents of the PtDA. This group offered support on navigability issues and how to develop the structure of the PtDA, acting as a review group in the testing of a series of PtDA prototypes.

#### Technical team

Constituted by designers, web developers and quality assurance specialists, (n = 4), this group provided technical and design support to the development of pilot testing PtDA and was coordinated and in constant communication with the Project Management Group.

### Pilot testing of the PtDA

#### First draft

The first draft version of the digital PtDA (developed and structure of content) was developed by Project Management Group and the Stakeholder consultation group, and presentation designs and technical issues were discussed with the technical team, until the first web-based PtDA prototype was decided on.

#### Second draft: field-testing

The second version of the digital PtDA was reviewed by patients with a current diagnosis of GAD. They evaluated its acceptability in terms of content-related dimensions (i.e., information quantity and clarity, things learned, willingness to ask their healthcare providers questions), format issues (i.e., ease of navigation, visual appealing, entertaining) and global appraisal (general assessment, usefulness to choose treatment and recommendations to other patients). All participant patients with GAD reviewed the online module of the digital PtDA and subsequently, completed the assessment questionnaire. Following the methodology proposed by Turner et al. [35], the researchers of the study developed the questionnaire items to assess the acceptability of the content of the PtDA, which was specifically designed for the project. This questionnaire included content-related items assessed on a 7-point Likert scale (from “too little information” to “too much information”) and one open question about what information may be missing from this PtDA. The items related to “clarity” were also assessed on a 7-point Likert scale (from “not understood at all” to “perfectly understood”). Additionally, there was one question asking if there were any words that were difficult to understand or needed extra clarification; with answers ranging from “no”, to “it seems to be quite clear”, or “yes, it would be necessary to add some extra definition”. Another question was related to the degree of understanding of the information on the showed bar charts (referring to how much each treatment reduces anxiety and improves quality of life) and the information directly stated in the text. Two questions were related to learning about treatments for GAD-7 and their risks and side effects on a 7-point Likert scale (from “I have learned nothing” to “I have learned many things”). The last question in this block asked whether “you would ask your health professional about things you have seen or read in the PtDA that you did not know before”, on a 7-point Likert scale (from “I would have nothing to ask” to “I would ask many questions”). Other questions related to format, navigation and global appraisement were assessed on a 5-point Likert scale (from “strongly disagree” to “strongly agree”). The final question was related to “whether or not recommend this PtDA to others”; also assessed on a 7-point scale (from “I would not recommend it at all” to “I would absolutely recommend it").

#### Final version of the PtDA

Results from field testing including all open comments were reviewed by the Project Management Group, with the support of the technical team; serving to improve content-related dimensions and format and navigation, which led to the final version PtDA*.*

The effectiveness of the PtDA will be assessed in a randomized-controlled trial with a larger sample of primary care patients with GAD. This step is currently underway (registration number: NCT04364958). Once the trial is completed, the PtDA will be refined based on the feedback obtained, and hosted on the platform PyDeSalud (www.pydesalud.com), for patients with GAD or clinicians wishing to implement an SDM process to promote person-centered care during clinical encounters.

### Participants, recruitment, data collection and data analysis

The study was conducted on a Spanish population formed by adults (18 years of age or older) with a current diagnosis of GAD in electronic medical records (EMR), based on the International Statistical Classification of Diseases (ICD) [36]. In order to reduce selection bias by health professionals, any patients with a current diagnosis of GAD in EMR could be a potential candidate. Patients with a diagnosis other than GAD were excluded. Different researchers provided a brief demonstration on how to use the PtDA in clinical practice, in a health center. Physicians contacted their patients with GAD and invited them to participate in the PtDA review. Patients who agreed to participate were then contacted by phone by a researcher that used a semi-structured interview to collect sociodemographic and clinical data, including the verbal informed consent. A link to the PtDA was then sent by e-mail to the patient. After they had reviewed it, a second telephone call was conducted in order to assess acceptability. Descriptive analyses (percentages, means and standard deviations) for all the included variables were performed. The scale of acceptability can be found in Additional file 1.

## Results

### Development and review of the first PtDA draft

The first PtDA version was developed according to: (1) the scientific evidence extracted from the CPG, including information about the disorder and its pharmacological and psychological treatments, and (2) the information obtained in the three studies carried out to assess patients’ experiences and preferences [19, 33, 34]. This version was reviewed by the Project Management Group and Stakeholder consultation group, that provided feedback through an iterative process. After which, the subsequent PtDA version was discussed with the technical team.

### Development and review of the second PtDA draft: main results on acceptability

Eleven patients (63.4% female) with GAD diagnosis assessed PtDA acceptability (Table 1). Mean age was 45.7 years (range 29–58) and mean time since diagnosis was 12.0 years (range 1–39). The detailed sociodemographic and clinical data of participants are shown in Table 1.

**Table 1:** Sociodemographic and clinical data of participants in the PtDA pilot testing

Gender *(n, %)*	
Male	4 (36.7)
Female	7 (63.4)
Mean, age *(SD)*	45.7 (11.9)
Education (*n, %)*	
Primary studies	1 (9.1)
Secondary studies	2 (18.1)
University studies	6 (54.6)
Postgraduate studies (master's, doctorate)	2 (18.1)
Mean time since diagnosis, year *(n, SD)*	12.0 (12.6)
Current treatment *(n, %)*	
None	3 (27.3)
Pharmacological	3 (27.3)
Psychological	1 (9.1)
Both	4 (36.4)

**SD**: Standard deviation

**n**: number of participants

**%**: percentage of participants

The results obtained in the different sections of the questionnaire are shown in Table 2.

**Table 2:** Acceptability of the PtDA

Content-related	
What do you think about the AMOUNT of information in the PtDA?	
(1–2) Little information (n, %)	0 (0.0)
(3–5) Acceptable information (n, %)	5 (45.5)
(6–7) Too much information (n, %)	6 (54.6)
In general, what information do you think may be MISSING from the tool that you would like to know? Do you have any other comments you would like to make about your impression of the PtDA? (open-ended question)	
I don't think anything is missing (n, %)	3 (27.3)
Yes, something is missing (n, %)	8 (72.7)
*-What to do with all this information after viewing the PtDA*	2 (18.2)
*-Aspects related to the PtDA dynamism*	1 (9.1)
*-Aspects related to language*	1 (9.1)
*-About other psychological and pharmacological treatments*	1 (9.1)
*-Information related to the causes of anxiety*	2 (18.2)
*-Side effects of long-term pharmacological treatments and help for family members*	1 (9.1)
What do you think about the CLARITY of the information in the PtDA?	
(1–2) Not understood at all (n, %)	0 (0.0)
(3–5) Some is understood (n, %)	4 (36.4)
(6–7) It is perfectly understood (n, %)	7 (63.6)
Are there any word(s) that you find difficult to understand or would need to be defined? Which ones?	
No, it seems to be quite clear (n, %)	7 (63.6)
Yes, it would be necessary to add some extra definition (n, %)	4 (36.4)
In the information referring to how much each of the treatments reduces anxiety and improves quality of life, do you think that the text and the graph shown are clearly undersandable or do you think it could be interpreted differently?	
Do not know (n, %)	1 (9.1)
No (n, %)	4 (36.4)
Yes (n, %)	6 (54.6)
Do you think you have learned new things about TREATMENTS for GAD?	
(1–2) I have learned nothing (n, %)	3 (27.3)
(3–5) I have learned something (n, %)	4 (36.4)
(6–7) I have learned many things (n, %)	4 (36.4)
Do you think you have learned anything new about the RISKS AND SIDE EFFECTS of treatments for GAD?	
(1–2) I have learned nothing (n, %)	3 (27.3)
(3–5) I have learned something (n, %)	3 (27.3)
(6–7) I have learned many things (n, %)	5 (45.5)
Do you think you would ASK YOUR HEALTH CARE PROFESSIONAL about things you have seen and read in the PtDA that you didn't know before?	
(1–2) I would have nothing to ask (n, %)	1 (9.1)
(3–5) I would ask some questions (n, %)	7 (63.6)
(6–7) I would ask many questions (n, %)	3 (27.3)
*Format and navigation*	
Do you find this help tool easy to use?	
(1) Strongly disagree (n, %)	0 (0)
(2) Quite disagree (n, %)	0 (0)
(3) Neither agree nor disagree (n, %)	4 (36.4)
(4) Quite agree (n, %)	2 (18.2)
(5) Strongly agree (n, %)	5 (45.5)
Do you think this help tool is visually appealing?	
(1) Strongly disagree (n, %)	0 (0)
(2) Quite disagree (n, %)	1 (9.1)
(3) Neither agree nor disagree (n, %)	3 (27.3)
(4) Quite agree (n, %)	1 (9.1)
(5) Strongly agree (n, %)	6 (54.6)
Do you think that this help tool is entertaining?	
(1) Strongly disagree (n, %)	0 (0)
(2) Quite disagree (n, %)	1 (9.1)
(3) Neither agree nor disagree (n, %)	1 (9.1)
(4) Quite agree (n, %)	3 (27.3)
(5) Strongly agree (n, %)	6 (54.6)
*Overall appraisal*	
Do you think that this tool is useful?	
(1) Strongly disagree (n, %)	0 (0)
(2) Quite disagree (n, %)	0 (0)
(3) Neither agree nor disagree (n, %)	3 (27.3)
(4) Quite agree (n, %)	2 (18.2)
(5) Strongly agree (n, %)	6 (54.6)
If you had to choose a treatment for your GAD, would you use this PtDA to help you?	
(1) Strongly disagree (n, %)	0 (0)
(2) Quite disagree (n, %)	1 (9.1)
(3) Neither agree nor disagree (n, %)	2 (18.2)
(4) Quite agree (n, %)	3 (27.6)
(5) Strongly agree (n, %)	5 (45.5)
If you had a friend with generalized anxiety, would you RECOMMEND this web PtDA?	
(1–3) Would not recommend it at all (n, %)	0 (0.0)
(4–5) Would recommend it in part (n, %)	3 (27.3)
(6–7) Would recommend it absolutely (n, %)	8 (72.7)

(*) Scale developed and adapted by the researchers following the methodology proposed by Turner et al. [28]

GAD: Generalized Anxiety Disorder

PtDA: Patient Decision Aids

### Content-related dimensions

Regarding the *quantity of information* included in the PtDA, five participants (45.5%) thought that it was acceptable, whereas six (54.6%) said that it was too much information. An open question was included about information that may be missing in the PtDA or that the person would like to know. Three participants considered that nothing was missing (27.3%), and the remaining mentioned the following topics: (1) “*What to do with all that information after seeing the tool”; (2) “Aspects related to the tool's dynamism”; (3) “Aspects related to language”; (4) “About other psychological and pharmacological treatments”; (5) “Information related to the causes of anxiety”; and (6) “Side effects of long-term drug treatments and help for family members”.*

Regarding the *clarity of information*, seven (63.6%) stated that the information was perfectly clear, and four (36.4%) said that some aspects were not well explained and some extra definitions were needed. Similarly, four participants thought that the bar charts showing the mean average improvement in anxiety and quality of life were not clearly understandable, whereas six (54.6%) thought that they were.

Three participants (27.3%) said that they had not learned anything new about GAD treatments and their risk of adverse effects, whereas eight (82.7%) thought that they had learned something or a lot. Ten participants said that they would ask their healthcare provider some (3, 27.3%) or a lot (7, 63.6%) of the questions about information they had read in the PtDA.

### Format and navigation

Seven participants agreed (18.2%) or strongly agreed (45.5%) that the PtDA was easy to use, whereas four (36.4%) did not agree or disagreed. Seven participants (63.6%) considered it visually appealing, whereas one disagreed and three (27.3%) did not agree or disagree. Nine participants (81.9%) agreed or strongly agreed that the PtDA was entertaining.

### Overall appraisal

Eight participants (72.7%) considered that the PtDA was useful, and three neither agreed nor disagreed. Likewise, eight participants agreed or strongly agreed that the PtDA could help them to choose a treatment for GAD, one disagreed and two neither agreed nor disagreed. Finally, eight participants (72.7%) would absolutely recommend the PtDA, whereas three (27.3%) would recommend it in part.

### Final version PtDA

**Fig. 2:**
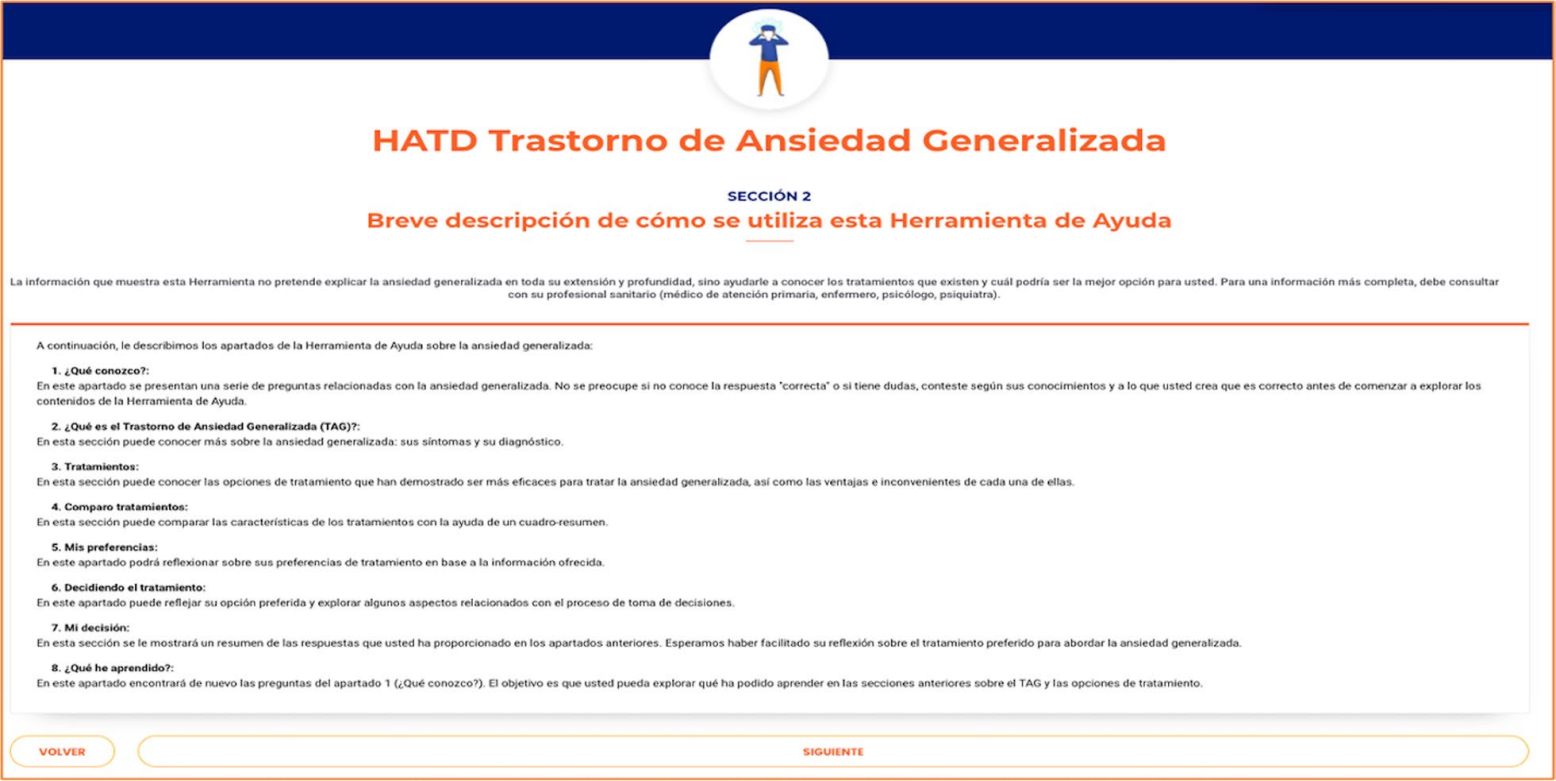
A brief summary of the PtDA contents

**Fig. 3:**
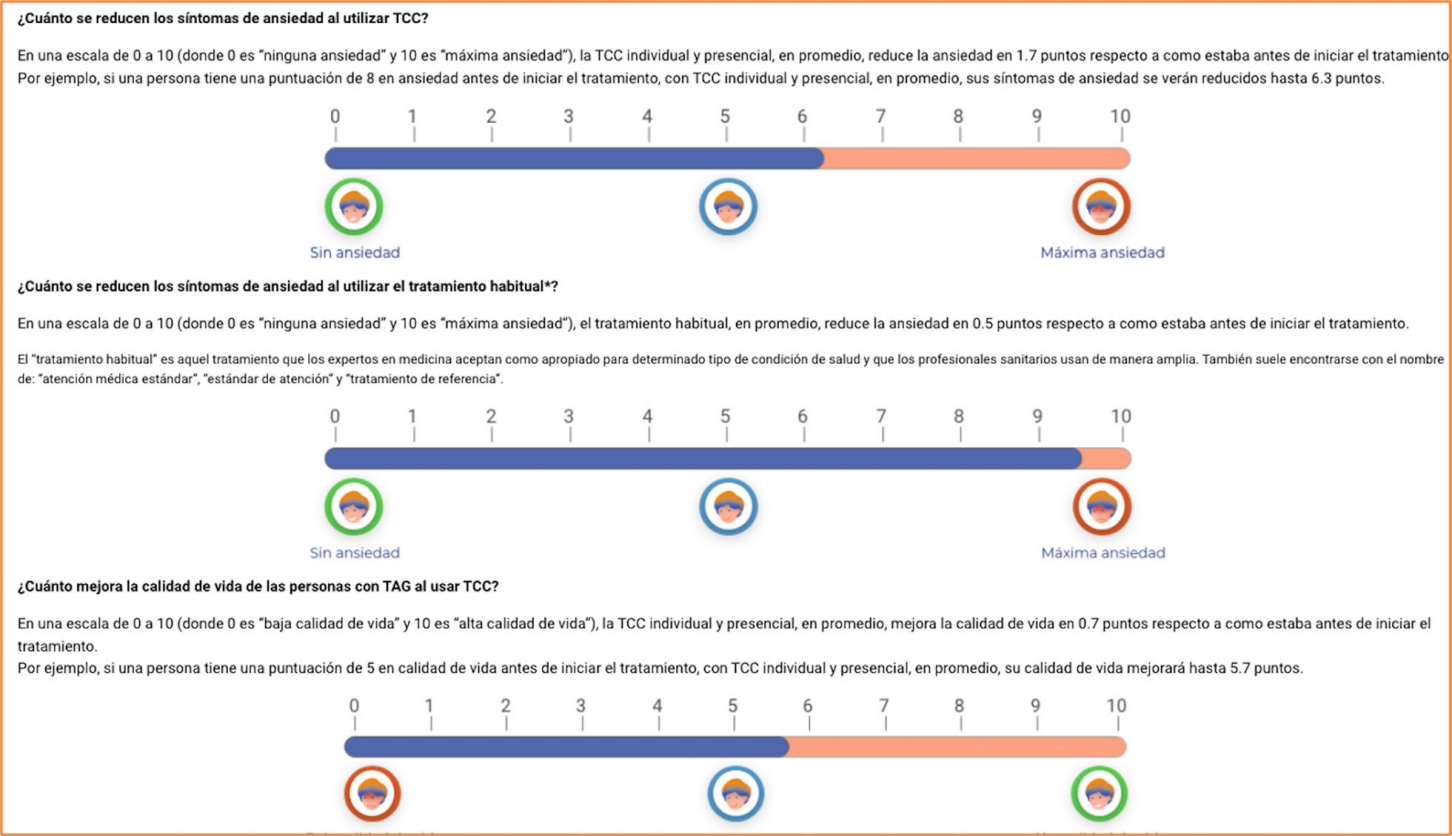
The description of each treatment option

**Fig. 4:**
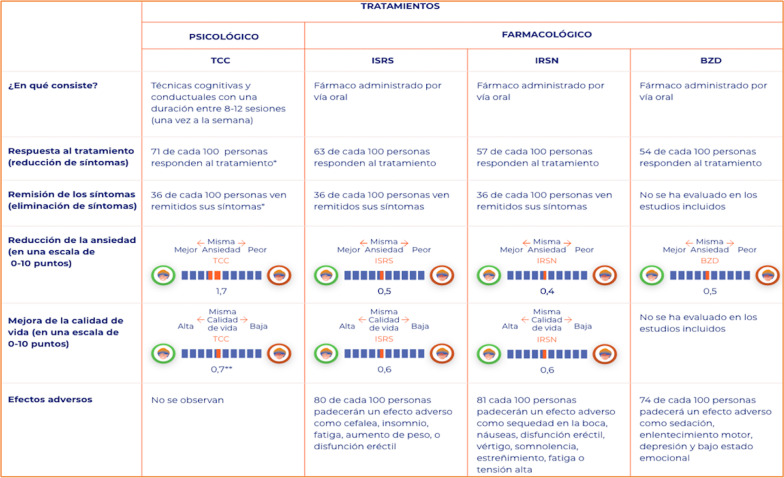
Summary table with a short description of each treatment’s for GAD

After the acceptability testing, changes in the PtDA were made based on the suggestions of the participants. The final PtDA was composed of the following sections (each one including information in both text and audio format):

1. A brief summary of the PtDA contents (screenshot shown in Fig. 2).
2. Questionnaire to assess knowledge about GAD and its treatments.
3. Description of the GAD (definition, symptoms, diagnosis, and etiology: psychological, environmental, socio-economic, genetic and neurobiological factors).
4. Available treatments for GAD: psychological treatment including cognitive-behavioral therapy and pharmacological treatment including SSRIs, SNRIs, and benzodiazepines.
5. Description of treatment option, including: (a) what the treatment consists of; (b) percentage of patients who respond to treatment; (c) percentage of patients who go into remission; (d) mean improvement in anxiety and mental-related quality of life on a 0–10 scale (screenshot shown in Fig. 3); and (e) mild and serious adverse effects of each treatment.
6. Summary table with a short description of each treatment’s characteristics and the remaining information previously shown, so that the patient can easily compare them in terms of benefits and risks of the PtDA (screenshot shown in Fig. 4). In each section, the incorporation of audio was available if the participant wanted to listen to it.
7. Finally, at the end of the PtDA, the following questionnaires were included: the Decisional Conflict Scale, that evaluates the level of the patient’s subjective knowledge, perceived support and uncertainty when they are confronted with a medical decision; knowledge about the disorder and treatment alternatives (also presented in Sect. 2), and a scale about treatment preference, assessed with one item with four response alternatives: (pharmacological treatment, psychological treatment, combined pharmacological and psychological treatment or unsure). These questionnaires are part of the outcome measures of the ongoing trial [37].

## Discussion

SDM promotes informed and rational deliberation between patients and healthcare professionals in order to choose among different treatment options, based on scientific evidence about their potential benefits and risks, and incorporating patients’ preferences and values into the final decision. Due to population health literacy limitations, communicating available scientific evidence for decision-making between patients and healthcare professionals remain as a challenge [38], and PtDAs may help patients to make better informed decisions, congruent with their preferences. The present article describes the development process and acceptability testing of a PtDA for patients with GAD, efficiently developed in the context of a CPG updating for this population. SDM requires that evidence is conveyed to the patient so that they can easily understand and compare their options. Research about the use of simple visual displays such as icon arrays or bar charts to communicate the risks associated with interventions improves the patients' ability to understand [38, 39] and even the quality of the patient-healthcare professional interaction [40]. Approximately half of the participants indicated that the information on treatment effects, with icon arrays and bar charts, is understandable.

Seeking information about the causes of illness and available treatments is a common aspect in chronic mental illnesses, such as in bipolar disorder [41], mood disorders [42], and specifically in GAD [19]. The results of the present study continue to reinforce what has been found in the literature thus far, with 72.8% of the participants stating that the PtDA was helpful in terms of learning. Pilot studies evaluating the acceptability of PtDA in patients with depression [43] and bipolar disorder [44] showed good acceptability in terms of usefulness and ease of use.

Previous investigation shows that most patients with mental disorders want to participate in the decisions about their treatment [45–47]. However, patient participation in research and development of PtDAs is almost absent in GAD. The authors have previously shown in a small sample that most GAD patients prefer an active or collaborative role in decision making, and only about half see their preferences fulfilled [19]. The present PtDA aims to help fill this gap, promoting patients’ knowledge and discussion with their healthcare providers. The ongoing trial on the PtDA’s effectiveness [37] might confirm whether improvements in decision-making related variables are similar to those observed in other mental disorders [48, 49] or whether the GAD population shows its own specificities [18, 23].

The development of the CPG is beginning to incorporate patients’ values and preferences to embrace a more person-centered approach in the decision-making process [50]. Moreover, the GRADE group developed the evidence to decision (EtD) framework, contributing to the development of CPGs in a structured and transparent manner [51]. Initiatives as the DECIDE project [52] the MAGIC project or PtDAs [53, 54] or App MAGICapp (www.magicapp.org) [55]. may help to incorporate the best available evidence inside and outside the practice. One of the advantages of these initiatives is that the development of CPG recommendations is based on a rigorous process, within the Evidence to Decision frameworks [56]. This process allows the identification of the most SDM sensitive recommendations from the early stages of the CPG development, facilitating the task to decide when to develop a parallel PtDAs [28, 57]. Heen et al. [58] showed that PtDA linked to CPG evidence summaries induced a positive shift in consultation habits towards SDM.

PtDA are often not based on current evidence or are rapidly outdated, at least in part because a rigorous systematic review is needed for each relevant clinical outcome, and such reviews are often unavailable. A recent assessment found that although around two thirds of PtDAs are based on systematic reviews or guidelines, many of these sources are of questionable quality, and only 5% of aids included an “expiry date” or a stated policy about updating [59].

### Limitations

An important limitation of this study is the low number of patients included in the development of the first prototype (n = 2), partly due to the emergence of the COVID-19 pandemic. In addition, the sample of patients who assessed the acceptability of the PtDA was small and may not be representative of the patients with GAD seen in clinical practice. Most of them have university education, and therefore uncertainty persists about the PtDA acceptability for people with a lower level of education or health literacy. The incorporation of audio tries to facilitate its use by people less accustomed to reading. Recent research suggests that psychiatric patients (with several cognitive deficits) are capable of using the PtDA and can benefit from doing so [60, 61]. A recent systematic review reported that for socially disadvantaged patients in particular, with a lower level of digital health literacy, the use of PtDA leads to better health outcomes [62]. Future projects should examine whether the level of digital health literacy is a key factor that could influence the health outcomes of patients with GAD. The sample may also be biased in terms of motivation to participate in the study, compared to the general GAD population. Regarding the PtDA, no specific information for family members is included at the moment, and treatments are restricted to those with greater evidence about their effectiveness.

Another highlight of the research is the limitation of adequately translating evidence from CPG recommendations [63]. One of these limitations may be that the guidelines for PtDA development may not be sufficient to ensure that developers select the best available evidence and present the evidence appropriately. It seems to be common to present biased low-certainty evidence in PtDAs, which may lead to inadvertent support for low-value care. Zadro et al. [63] support the need for: (1) reporting only on studies based on high-quality evidence (randomized clinical trials or systematic reviews); (2) presenting estimates of the benefits and risks of treatments to minimize patients' bias toward a particular intervention, reporting quantitative estimates of inter-group effects, including (where possible) outcomes of the "no treatment" or "wait and see" option, and recognizing the level of evidence (e.g., using a star system in which five stars indicate high-certainty evidence)" [63].

Taking into account these limitations, to the best of the authors’ knowledge, the PtDA described here is the first prototype for patients with GAD. Strengths of the study include the adoption of a standardized methodology and criteria for the development of PtDAs [20], including a rigorous evidence search and synthesis process, in the context of a CPG update.

## Conclusions

The PtDA seems acceptable for GAD patients. An evaluation of its effectiveness to improve patients’ decision-related variables is needed, as well as its effects on the SDM process during the clinical encounter. The present study offers a starting point for incorporating decision aids for GAD patients, which are currently non-existent.

## Supplementary Information

**Additional file 1:** Sociodemographic and clinical data.

## Data Availability

All data generated or analyzed during this study are included in this published article [and its additional files].

## Abbreviations

CPG: Clinical Practice Guidelines
DCS: Decisional Conflict Scale
EMR: Electronic Medical Records
EtD: Evidence to Decision
GAD: Generalized Anxiety Disorder
GRADE: Grading of Recommendations, Assessment, Development and Evaluation
ICD: International Statistical Classification of Diseases
IPDAS: International patient decision aids standards
PtDA: Patient Decision Aid
SDM: Shared Decision Making
SNRIs: Serotonin and Noradrenaline Reuptake Inhibitors
SSRIs: Selective Inhibitors of Serotonin reuptake
WHO: World Health Organization

## References

[1] World Health Organization (WHO). Mental health in the workplace. 2017. https://www.who.int/teams/mental-health-and-substance-use/promotion-prevention/mental-health-in-the-workplace. Accessed 1 Jan 2022.

[2] World Health Organization (WHO). The World Health Report 2013-Research for Universal Health Coverage. WHO Press, Geneva; 2013.

[3] Ruscio AM, Hallion LS, Lim CCW, Aguilar-Gaxiola S, Al-Hamzawi A, Alonso J (2017). Cross-sectional comparison of the epidemiology of DSM-5 generalized anxiety disorder across the globe. JAMA Psychiat.

[4] Health Quality Ontario (2017). Psychotherapy for major depressive disorder and generalized anxiety disorder: a health technology assessment. Ont Health Technol Assess Ser.

[5] Caballero L, Bobes J, Vilardaga I, Rejas J. Prevalencia clinica y motivo de consulta en pacientes con trastorno de ansiedad generalizada atendidos en consultas ambulatorias de psiquiatria en España. Resultados del estudio LIGANDO. Actas Esp Psiquiatr. 2009;37:17–20. 18815907

[6] Rovira J, Albarracin G, Salvador L, Rejas J, Sánchez-Iriso E, Cabasés JM (2012). The cost of generalized anxiety disorder in primary care settings: results of the ANCORA study. Community Ment Health J.

[7] National Institute for Health and Care Excellence (NICE). Generalised anxiety disorder and panic disorder in adults: management. https://www.nice.org.uk/guidance/cg113/chapter/1-Guidance#stepped-care-for-people-with-gad. Accessed 6 Feb 2022. 31961629

[8] Fumero A, Peñate W, Oyanadel C, Porter B. The effectiveness of mindfulness-based interventions on anxiety disorders. a systematic meta-review. Eur J Investig Heal Psychol Educ. 2020;10:704–19. 10.3390/ejihpe10030052 PMC8314302 34542506

[9] Makoul G, Clayman ML (2006). An integrative model of shared decision making in medical encounters. Patient Educ Couns.

[10] Charles C, Gafni A, Whelan T (1997). Shared decision-making in the medical encounter: what does it mean? (or it takes at least two to tango). Soc Sci Med.

[11] Elwyn G, Durand MA, Song J, Aarts J, Barr PJ, Berger Z, et al. A three-talk model for shared decision making: multistage consultation process. BMJ. 2017;j4891. 10.1136/bmj.j4891 PMC5683042 29109079

[12] Elwyn G, Frosch D, Thomson R, Joseph-Williams N, Lloyd A, Kinnersley P (2012). Shared decision making: a model for clinical practice. J Gen Intern Med.

[13] Scholl I, Loon MK, Sepucha K, Elwyn G, Légaré F, Härter M (2011). Measurement of shared decision making—a review of instruments. Z Evid Fortbild Qual Gesundhwes.

[14] Toupin-April K, Décary S, de Wit M, Meara A, Barton JL, Fraenkel L (2021). Endorsement of the OMERACT core domain set for shared decision making interventions in rheumatology trials: results from a multi-stepped consensus-building approach. Semin Arthritis Rheum.

[15] Niburski K, Guadagno E, Abbasgholizadeh-Rahimi S, Poenaru D (2020). Shared decision making in surgery: a meta-analysis of existing literature. Patient Patient-Centered Outcomes Res.

[16] Stacey D, Légaré F, Lewis K, Barry MJ, Bennett CL, Eden KB, et al. Decision aids for people facing health treatment or screening decisions. Cochrane Database Syst Rev. 2017;2017. 10.1002/14651858.CD001431.pub5 PMC6478132 28402085

[17] Politi MC, Clark MA, Ombao H, Dizon D, Elwyn G (2011). Communicating uncertainty can lead to less decision satisfaction: a necessary cost of involving patients in shared decision making?. Heal Expect.

[18] van der Heiden C, Broeren S, Bannink R, Crezee K, Zanolie K (2019). Intolerance of uncertainty and decision making in generalized anxiety disorder patients. Psychiatry Res.

[19] Ramos-García V, Rivero-Santana A, Duarte-Díaz A, Perestelo-Pérez L, Peñate-Castro W, Álvarez-Pérez Y (2021). Shared decision-making and information needs among people with generalized anxiety disorder. Eur J Investig Heal Psychol Educ.

[20] Coulter A, Stilwell D, Kryworuchko J, Mullen PD, Ng CJ, van der Weijden T (2013). A systematic development process for patient decision aids. BMC Med Inform Decis Mak.

[21] Vincent Y-M, Frachon A, Buffeteau C, Conort G (2021). Construction of a patient decision aid for the treatment of uncomplicated urinary tract infection in primary care. BMC Fam Pract.

[22] Nota I, Drossaert CHC, Melissant HC, Taal E, Vonkeman HE, Haagsma CJ (2017). Development of a web-based patient decision aid for initiating disease modifying anti-rheumatic drugs using user-centred design methods. BMC Med Inform Decis Mak.

[23] Marshall T, Stellick C, Abba-Aji A, Lewanczuk R, Li X-M, Olson K (2021). The impact of shared decision-making on the treatment of anxiety and depressive disorders: systematic review. BJPsych Open.

[24] Agoritsas T, Heen AF, Brandt L, Alonso-Coello P, Kristiansen A, Akl EA (2015). Decision aids that really promote shared decision making: the pace quickens. BMJ.

[25] Perestelo Pérez L, Salcedo Fernández F, Toledo Chávarri A, Álvarez Pérez Y, Vivente Edo M, Abt Sacks A. Desarrollo de herramientas de ayuda para la toma de decisiones compartida derivadas de las recomendaciones de las guías de práctica clínica. Ministerio de Sanidad, Servicios Sociales e Igualdad. Servicio de Evaluación del Servicio Canario de la Salud. Informes de Evaluación de Tecnologías Sanitarias; 2017.

[26] GRADE Working Gr. Key DECIDE tools | DECIDE (2011–2015). https://www.decide-collaboration.eu/. Accessed 15 Jan 2022.

[27] Alonso Coello P, Álvarez Pérez Y, Bono Vega M, Gavín Benavent P, Perestelo Pérez L, Prieto Remón L, et al. Aplicación de las recomendaciones de las guías de práctica clínica a la toma de decisiones compartida. 2022.

[28] National Institute for Health and Care Excellence. NICE decision aids: process guide. 2018. https://www.nice.org.uk/Media/Default/About/what-we-do/our-programmes/nice-guidance/shared-decision-making/decision-aid-process-guide.pdf. Accessed 24 Feb 2022.

[29] Grupo de Trabajo de la Guia de Práctica Clínica para el Manejo de Pacientes con Trastornos de Ansiedad en Atención Primaria. Guía de Práctica Clínica para el Manejo de Pacientes con Trastornos de Ansiedad en Atención Primaria. Madrid. Plan Nacional para el SNS del MSC. Unidad de Evaluación de Tecnologías Sanitarias. Agencia Laín Entralgo: Guías de Práctica Clínica en el SNS: UETS No 2006/10; 2008.

[30] Roberge P, Normand-Lauzière F, Raymond I, Luc M, Tanguay-Bernard M-M, Duhoux A (2015). Generalized anxiety disorder in primary care: mental health services use and treatment adequacy. BMC Fam Pract.

[31] Elwyn G, Kreuwel I, Durand MA, Sivell S, Joseph-Williams N, Evans R (2011). How to develop web-based decision support interventions for patients: a process map. Patient Educ Couns.

[32] Guyatt GH, Oxman AD, Vist GE, Kunz R, Falck-Ytter Y, Alonso-Coello P (2008). GRADE: an emerging consensus on rating quality of evidence and strength of recommendations. BMJ.

[33] Toledo-Chávarri A, Ramos-García V, Torres-Castaño A, Trujillo-Martín MM, Peñate Castro W, Del Cura-Castro I (2020). Framing the process in the implementation of care for people with generalized anxiety disorder in primary care: a qualitative evidence synthesis. BMC Fam Pract.

[34] Ramos-García V, Toledo-Chávarri A, Trujillo-Martín M, Pino-Sedeño T, Peñate-Castro W, Perestelo-Pérez L. Experiences, values and preferences of people with generalized anxiety disorder about their health care: Qualitativ. 2020. p. 331–46.

[35] Turner M, Kitchenham B, Brereton P, Charters S, Budgen D (2010). Does the technology acceptance model predict actual use? A systematic literature review. Inf Softw Technol.

[36] World Health Organization (WHO). International Classification of Diseases, 11th Revision (ICD-11). 2019. https://www.who.int/classifications/icd/en/. Accessed 31 Jan 2022.

[37] Perestelo-Pérez L, Rivero-Santana A, Ramos-García V, Álvarez-Pérez Y, Duarte-Díaz A, Torres-Castaño A (2020). Effectiveness of a web-based decision aid for patients with generalised anxiety disorder: a protocol for a randomised controlled trial. BMJ Open.

[38] Trevena LJ, Zikmund-Fisher BJ, Edwards A, Gaissmaier W, Galesic M, Han PK (2013). Presenting quantitative information about decision outcomes: a risk communication primer for patient decision aid developers. BMC Med Inform Decis Mak.

[39] Bonner C, Trevena LJ, Gaissmaier W, Han PKJ, Okan Y, Ozanne E (2021). Current best practice for presenting probabilities in patient decision aids: fundamental principles. Med Decis Mak.

[40] Spiegelhalter D, Pearson M, Short I. Visualizing Uncertainty About the Future. Science (80). 2011;333:1393–400. 10.1126/science.1191181 21903802

[41] Conell J, Bauer R, Glenn T, Alda M, Ardau R, Baune BT (2016). Online information seeking by patients with bipolar disorder: results from an international multisite survey. Int J Bipolar Disord.

[42] Liebherz S, Härter M, Dirmaier J, Tlach L (2015). Information and decision-making needs among people with anxiety disorders: results of an online survey. Patient Patient Centered Outcomes Res.

[43] Barr PJ, Forcino RC, Dannenberg MD, Mishra M, Turner E, Zisman-Ilani Y (2019). Healthcare options for people experiencing depression (HOPE*D): the development and pilot testing of an encounter-based decision aid for use in primary care. BMJ Open.

[44] Fisher A, Keast R, Costa D, Sharpe L, Manicavasagar V, Anderson J (2020). Improving treatment decision-making in bipolar II disorder: a phase II randomised controlled trial of an online patient decision-aid. BMC Psychiatry.

[45] Puschner B, Becker T, Mayer B, Jordan H, Maj M, Fiorillo A (2016). Clinical decision making and outcome in the routine care of people with severe mental illness across Europe (CEDAR). Epidemiol Psychiatr Sci.

[46] Mundal I, Lara-Cabrera ML, Betancort M, De las Cuevas C. Exploring patterns in psychiatric outpatients’ preferences for involvement in decision-making: a latent class analysis approach. BMC Psychiatry. 2021;21:133. 10.1186/s12888-021-03137-x PMC7937224 33676452

[47] Burns L, da Silva AL, John A (2021). Shared decision-making preferences in mental health: does age matter? A systematic review. J Ment Heal.

[48] Shillington AC, Langenecker SA, Shelton RC, Foxworth P, Allen L, Rhodes M (2020). Development of a patient decision aid for treatment resistant depression. J Affect Disord.

[49] Perestelo-Perez L, Rivero-Santana A, Sanchez-Afonso JA, Perez-Ramos J, Castellano-Fuentes CL, Sepucha K (2017). Effectiveness of a decision aid for patients with depression: a randomized controlled trial. Heal Expect.

[50] van der Weijden T, Boivin A, Burgers J, Schünemann HJ, Elwyn G (2012). Clinical practice guidelines and patient decision aids. An inevitable relationship. J Clin Epidemiol.

[51] Alonso-Coello P, Schünemann HJ, Moberg J, Brignardello-Petersen R, Akl EA, Davoli M, et al. Marcos GRADE de la evidencia a la decisión (EtD): un enfoque sistemático y transparente para tomar decisiones sanitarias bien informadas. 1 Introducción. Gac Sanit. 2018;32:166e1–16e10 10.1016/j.gaceta.2017.02.010 28822594

[52] Treweek S, Oxman AD, Alderson P, Bossuyt PM, Brandt L, Brożek J (2013). Developing and evaluating communication strategies to support informed decisions and practice based on evidence (DECIDE): protocol and preliminary results. Implement Sci.

[53] Garcia-Retamero R, Cokely ET (2013). Communicating health risks with visual aids. Curr Dir Psychol Sci.

[54] Garcia-Retamero R, Cokely ET, Anderson BL, Schulkin J (2014). Using visual aids to help people with low numeracy make better decisions. Numerical reasoning in judgments and decision making about health.

[55] Kristiansen A, Brandt L, Alonso-Coello P, Agoritsas T, Akl EA, Conboy T (2015). Development of a novel, multilayered presentation format for clinical practice guidelines. Chest.

[56] Schünemann H, Brożek J, Guyatt G, Oxman A editors. GRADE handbook for grading quality of evidence and strength of recommendations: Updated October 2013 [Internet]. The GRADE Working Group; 2013. guidelinedevelopment.org/handbook. Accessed 24 Feb 2021.

[57] Tool for guidelines and shared decision making in practice. Hilbink M, Ouwens M, Kool T. De HARING-tools. IQ Healthcare; 2013.

[58] Heen AF, Vandvik PO, Brandt L, Achille F, Guyatt GH, Akl EA (2021). Decision aids linked to evidence summaries and clinical practice guidelines: results from user-testing in clinical encounters. BMC Med Inform Decis Mak.

[59] Montori VM, LeBlanc A, Buchholz A, Stilwell DL, Tsapas A (2013). Basing information on comprehensive, critically appraised, and up-to-date syntheses of the scientific evidence: a quality dimension of the International Patient Decision Aid Standards. BMC Med Inform Decis Mak.

[60] Drake RE, Cimpean D, Torrey WC (2009). Shared decision making in mental health: prospects for personalized medicine. Dialogues Clin Neurosci.

[61] Zisman-Ilani Y, Shern D, Deegan P, Kreyenbuhl J, Dixon L, Drake R (2018). Continue, adjust, or stop antipsychotic medication: developing and user testing an encounter decision aid for people with first-episode and long-term psychosis. BMC Psychiatry.

[62] Yen RW, Smith J, Engel J, Muscat DM, Smith SK, Mancini J (2021). A systematic review and meta-analysis of patient decision aids for socially disadvantaged populations: update from the international patient decision aid standards (IDPAS). Med Decis Mak.

[63] Zadro JR, Traeger AC, Décary S, O’Keeffe M (2021). Problem with patient decision aids. BMJ Evid Based Med.

[64] Grupo de Trabajo de la Guía de Práctica Clínica para el Manejo de Pacientes con Trastornos de Ansiedad en Atención Primaria. Guía de Práctica Clínica para el Manejo de Pacientes con Trastornos de Ansiedad en Atención Primaria. Madrid. Plan Nacional para el SNS del MSC. Unidad de Evaluación de Tecnologías Sanitarias. Agencia Laín Entralgo: Guías de Práctica Clínica en el SNS: UETS No 2006/10; 2018.

