# Correction to: Decision aids linked to the recommendations in clinical practice guidelines: results of the acceptability of a decision aid for patients with generalized anxiety disorder

**DOI:** 10.1186/s12911-022-01930-6

**Published:** 2022-07-18

**Authors:** Vanesa Ramos-García, Lilisbeth Perestelo-Pérez, Amado Rivero-Santana, Wenceslao Peñate-Castro, Andrea Duarte-Díaz, Yolanda Álvarez-Pérez, María del Mar Trujillo-Martín, María Isabel del Cura-González, Pedro Serrano-Aguilar

**Affiliations:** 1 Canary Islands Health Research Institute Foundation, Tenerife, Spain; 2 Facultad de Ciencias de la Salud - Sección de Psicología, University of La Laguna (ULL), Tenerife, Spain; 3 Evaluation Unit (SESCS), Canary Islands Health Service (SCS), Tenerife, Spain; 4 Research Network on Health Services in Chronic Diseases (REDISSEC), Tenerife, Spain; 5 Network for Research on Chronicity, Primary Care, and Health Promotion (RICAPPS), Tenerife, Spain; 6 The Spanish Network of Agencies for Health Technology Assessment and Services of the National Health System (RedETS), Tenerife, Spain; 7 Research Network on Health Services in Chronic Diseases (REDISSEC), Madrid, Spain; 8 Network for Research on Chronicity, Primary Care, and Health Promotion (RICAPPS), Madrid, Spain; 9 Unidad de Apoyo a la Investigación, Gerencia Asistencial de Atención Primaria, Madrid, Spain; 10 Department Preventive Medicine and Public Health, University Rey Juan Carlos, Madrid, Spain; 11 Servicio de Evaluación del Servicio Canario de la Salud, Camino de Candelaria nº 44, Centro de Salud San Isidro-El Chorrillo 1ª planta, 38109 Tenerife, Spain

## Correction to: BMC Medical Informatics and Decision Making (2022) 22:171 https://doi.org/10.1186/s12911-022-01899-2

Following publication of the original article [1], it was reported that the Given- and Family Names of the authors were transposed. The correct author list is given in this Correction article and the original article [1] has been updated.
