# Supplementary material for: Decision aids linked to the recommendations in clinical practice guidelines: results of the acceptability of a decision aid for patients with generalized anxiety disorder
Source: BMC Med Inform Decis Mak. 2022 Jun 30;22:171. doi: 10.1186/s12911-022-01899-2 (PMC9243714; doi:10.1186/s12911-022-01899-2)
Supplement: Supplementary file 1 — Additional file 1: Sociodemographic and clinical data. [file 12911_2022_1899_MOESM1_ESM.docx]

**Additional file 1**

| 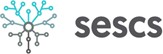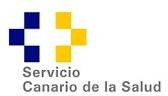  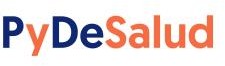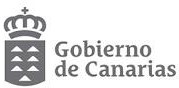 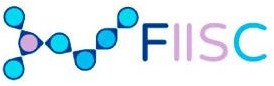 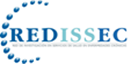  **DATOS SOCIODEMOGRÁFICOS Y CLÍNICOS**  Género   - Hombre - Mujer   Edad: ____  Estudios:   - Estudios primarios - Estudios secundarios - Estudios universitarios - Estudios de postgrado   Tiempo medio desde el diagnóstico (en años)______  Tratamiento actual   - Ninguno - Farmacológico - Psicológico - Ambos   **ESCALA DE ACEPTABILIDAD**   \| 1. **RELACIONADOS CON EL CONTENIDO**    1. **En una escala de 1 al 7 donde 1 es “muy poca información” y 7 es “mucha información”, ¿qué piensa usted sobre la cantidad de información en esta herramienta de ayuda?**    2. **En general, ¿qué información cree usted que podría faltar en esta herramienta y que a usted le hubiera gustado saber? ¿Tiene algún otro comentario que le gustaría hacer sobre tu impresión acerca de la herramienta de ayuda? (pregunta abierta)**       1. No creo que falte nada       2. Sí, creo que falta algo (indíquelo a continuación): ______________________________________________________________________________________________________________________________________________________________________________________________________    3. **En una escala de 1 al 7 donde 1 es “no se entiende nada” y 7 es “se entiende perfectamente” ¿Qué piensa usted sobre la CLARIDAD de la información en la herramienta de ayuda?**    4. **¿Hay alguna palabra o palabras que le hayan resultado difícil de entender y que cree que necesitan una definición extra? ¿cuáles?**        1. No, todo lo que he visto está bastante claro       2. Si, algunas de las palabras que necesitan una definición extra son:   ____________________________________________________________________________________________________________________________________________________________________________________________________________   - 1. **En la información referida a cuánto reduce la ansiedad y mejora la calidad de vida cada uno de los tratamientos, ¿crees que el texto y el gráfico mostrado son claramente entendibles o crees que podría interpretarse de otra manera?**   2. **En una escala de 1 al 7 donde 1 es “no he aprendido nada” y 7 es “he aprendido muchas cosas” ¿Cree usted que ha aprendido cosas nuevas sobre los TRATAMIENTOS para el TAG?**   3. **En una escala de 1 al 7 donde 1 es “no he aprendido nada” y 7 es “he aprendido muchas cosas” ¿Cree usted que ha aprendido cosas nuevas sobre los RIESGOS Y EFECTOS SECUNDARIOS DE LOS TRATAMIENTOS para el TAG?**   4. **En una escala de 1 al 7 donde 1 es “no tendría nada que preguntarle” y 7 es “le preguntaría muchas cosas” ¿Cree usted que le preguntaría a su profesional sanitario por cosas que ha visto y ha leído en esta herramienta de ayuda y que antes no sabía?**  1. **RELACIONADOS CON EL FORMATO Y LA NAVEGALIBILIDAD**   A continuación, le haré unas preguntas sobre los contenidos de la herramienta de ayuda para el trastorno de ansiedad generalizada. Por favor, conteste si está: Totalmente en desacuerdo (1); Bastante en desacuerdo (2); Ni de acuerdo ni en desacuerdo (3); Bastante de acuerdo (4); o Totalmente de acuerdo (5)  **2.1 ¿Cree usted que esta herramienta de ayuda es fácil de usar?**   - Totalmente en desacuerdo - Bastante en desacuerdo - Ni de acuerdo ni en desacuerdo - Bastante de acuerdo - Totalmente de acuerdo   **2.2 ¿Cree usted que esta herramienta de ayuda es visualmente atractiva?**   - Totalmente en desacuerdo - Bastante en desacuerdo - Ni de acuerdo ni en desacuerdo - Bastante de acuerdo - Totalmente de acuerdo   **2.2 ¿Cree usted que esta herramienta de ayuda es entretenida?**   - Totalmente en desacuerdo - Bastante en desacuerdo - Ni de acuerdo ni en desacuerdo - Bastante de acuerdo - Totalmente de acuerdo  1. **ASPECTOS GLOBALES**   **3.1. ¿Cree usted que esta herramienta de ayuda es útil?**   - Totalmente en desacuerdo - Bastante en desacuerdo - Ni de acuerdo ni en desacuerdo - Bastante de acuerdo - Totalmente de acuerdo   **3.2. Si tuviera que elegir un tratamiento para su TAG ¿utilizaría esta herramienta de ayuda?**   - Totalmente en desacuerdo - Bastante en desacuerdo - Ni de acuerdo ni en desacuerdo - Bastante de acuerdo - Totalmente de acuerdo   **3.2. Si tuviera algún amigo/a con TAG, ¿le recomendaría esta herramienta de ayuda?**   - Totalmente en desacuerdo - Bastante en desacuerdo - Ni de acuerdo ni en desacuerdo - Bastante de acuerdo - Totalmente de acuerdo \| \| --- \|   (*) Escala desarrollada y adaptada por los investigadores de este estudio siguiendo la metodología propuesta por Turner M, Kitchenham B, Brereton P, Charters S, Budgen D. Does the technology acceptance model predict actual use? A systematic literature review. Inf Softw Technol. 2010;52:463–79.  **TAG**: Trastorno de Ansiedad Generalizada |
| --- | --- |
